# NOTCH Single Nucleotide Polymorphisms in the Predisposition of Breast and Colorectal Cancers in Saudi Patients

**DOI:** 10.3389/pore.2021.616204

**Published:** 2021-04-09

**Authors:** Ibrahim O. Alanazi, Jilani Purusottapatnam Shaik, Narasimha Reddy Parine, Abdulrahman Al Naeem, Nahla A. Azzam, Majid A. Almadi, Abdulrahman M. Aljebreen, Othman Alharbi, Mohammad Saud Alanazi, Zahid Khan

**Affiliations:** ^1^ National Center for Biotechnology, King Abdulaziz City for Science and Technology, Riyadh, Kingdom of Saudi Arabia; ^2^ Genome Research Chair, Department of Biochemistry, College of Science, King Saud University, Riyadh, Kingdom of Saudi Arabia; ^3^ Basic Sciences Department, College of Science and Health Professions, King Saud bin Abdulaziz University for Health Sciences, Riyadh, Kingdom of Saudi Arabia; ^4^ Department of Women’s Imaging, King Fahad Medical City, Riyadh, Kingdom of Saudi Arabia; ^5^ College of Medicine, King Saud University, Riyadh, Kingdom of Saudi Arabia; ^6^ Division of Gastroenterology, Department of Medicine, King Khalid University Hospital, King Saud University, Riyadh, Kingdom of Saudi Arabia

**Keywords:** Notch, single nucleotide polymorphism, breast cancer, colorectal cancer, genetic screening marker

## Abstract

Breast cancer (BC) is a heterogeneous disease and is one of the most common malignancy affecting women worldwide while colorectal cancer (CRC) is estimated to be the third common cancer and second leading cause of cancer related death globally. Both BC and CRC involve multiple genetic and epigenetic alterations in genes belonging to various signaling pathways including NOTCH that has been implicated in the development of these cancers. We investigated four single nucleotide polymorphisms, each in genes encoding NOTCH1-4 receptors for their role in susceptibility to breast and colorectal cancers in Saudi population. In this case-control study, TaqMan genotypic analysis of rs3124591 in NOTCH1 and rs3820041 in NOTCH4 did not exhibit association with breast as well as colorectal cancers. However, a strong association of rs11249433 which is in close proximity to NOTCH2 was observed with breast cancer susceptibility especially with those having an early onset of the disease. Interestingly, the rs1043994 located in NOTCH3 showed gender preference and was found to be significantly associated with colorectal cancers in males. Validation of these findings in bigger populations of different ethnicities may prove beneficial in identifying rs11249433 and rs1043994 as genetic screening markers for early detection of breast and colorectal carcinomas, respectively.

## Introduction

Breast cancer (BC) is a heterogeneous and one of the common disease affecting women worldwide. BC accounts for 11.7 percent (2.261 million) of all new cancer cases and 6.9 percent (684,996) of all cancer deaths globally [1]. It is the most frequently diagnosed cancer in women living in Gulf Cooperation Council countries, including Saudi Arabia [2]. In Saudi Arabia, BC is ranked first among females with an estimated number of new cases to be 3,954 (29.0%) of the 13,632 total cancer cases in women, while BC related mortality was reported to be 1,095 (20.4%) of 5,376 cancer related deaths in 2020 [3]. About 15–30% of breast cancer cases might have underlying genetic transmission or heritable changes, however, these genetic alterations are not completely defined [4]. Genome-wide association studies (GWAS) have investigated common genetic variants and identified many genetic loci that are linked to the risk of BC [4]. Another leading human malignancy is colorectal cancer (CRC) ranking third in incidence (9.8%) and second in cancer related mortality (9.2%) in 2020 worldwide [1]. CRC is a heterogeneous disease involving the colon and rectum that harbors abnormalities in different molecular pathways. A number of epidemiological studies have shown that environmental factors and genetic susceptibility leads to the risk of developing CRC [5, 6]. In Saudi Arabia, the number of CRC incidence accounts for 3,924 cases (14.0%), while the number of CRC related deaths were estimated to be 1,964 (15.0%) for both genders. It is the leading malignancy in Saudi men with an estimated number of new cases to be 2702 (18.9%) in 2020 [3].

Several studies indicate that genetic variations are associated with risk of BC [7, 8] and play important role in CRC carcinogenesis and clinical outcome [9, 10]. Genetic variation includes the copy number variations (CNVs) that may involve sequence range between a few kilobases up to millions of bases, indels which include insertion/deletion of one or more nucleotides and single nucleotide polymorphisms (SNPs), which are the substitution of a single nucleotide along the DNA [11]. The location of these SNPs in or near genes that play important roles in signal transduction pathways, gene expression and regulation, control of cell cycle and genome stability, can profoundly affect the function of these genes. Several molecular mechanisms affecting signaling pathways including notch that regulates cell proliferation, differentiation and apoptosis have been reported in human cancers [12]. The NOTCH gene family consists of four receptors (NOTCH1, NOTCH2, NOTCH3, and NOTCH4). These receptors are expressed as heterodimers on the cell surface. Notch signaling plays an important role in several cellular processes including proliferation, epithelial cell polarity/adhesion and apoptosis. This signaling is activated by binding of notch ligand to epidermal growth factor (EGF) -like repeats on the neighboring cell notch receptor. Notch receptors have opposed functions in normal and cancerous breast tissues [13]. Aberrant notch signaling have been observed in various cancers including BC [14] and CRC [15]. This pathway has a significant role in the development of human breast cancer deduced from studies on mouse mammary tumor virus-induced cancer [14]. In CRC as well notch receptors are recognized to be highly oncogenic [16, 17]. *In vitro* overexpression of NOTCH1 has been implicated in colony formation, proliferation and tumorsphere formation of CRC cells. Additionally, NOTCH1 promotes growth and development of colon cancers *in vivo* [15].

Efforts are ongoing to identify reliable biomarkers for predicting human malignant neoplastic diseases. Genetic variation analysis has the potential to be utilized for screening and identifying novel prognosis genes. Single-nucleotide polymorphisms (SNPs) is one of the most common type of genetic variation that may serve as potential and distinctive genetic markers. In the present study, based on the associations with cancers from previous literature, we examined the influence of four SNPs, rs3124591, rs11249433, rs1043994, and rs3830041 which are located within or in close proximity of NOTCH1, NOTCH2, NOTCH3 and NOTCH4 genes, respectively on BC and CRC susceptibility by comparing the genotypic distribution of these SNPs in cancer cases to that of healthy subjects from Saudi Arabia.

## materials and methods

### Study Population

The study subjects included in this case-control study comprised of women with pathologically confirmed breast cancer and age-matched female controls of Saudi Arabian ethnicity without any history of cancer. The median age at the time of breast cancer diagnosis was 53 years. The number of cases and controls examined for each SNP were as follows: NOTCH1 rs3124591 (cases *n* = 190; controls *n* = 70), NOTCH2 related rs11249433 (cases *n* = 185; controls *n* = 133), NOTCH3 rs1043994 (cases *n* = 182; control *n* = 128), NOTCH4 rs3830041 (cases *n* = 186; controls *n* = 134). Pretreatment blood samples from breast cancer patients were collected at King Fahad Medical City, Riyadh.

The number of CRC cases and controls included in this study for each SNP were as follows: NOTCH1 rs3124591 (cases *n* = 96; controls *n* = 103), NOTCH2 related rs11249433 (cases *n* = 141; controls *n* = 139), NOTCH3 rs1043994 (cases *n* = 134; controls *n* = 139), NOTCH4 rs3830041 (cases *n* = 141; controls *n* = 139). The median age at diagnosis of CRC was 58 years. Age-matched controls were recruited after diagnostic exclusion of cancer and cancer-related diseases. Blood samples from CRC patients were taken prior to receiving treatment at King Khalid University Hospital, Riyadh.

### DNA Extraction

Ethylenediaminetetraacetic acid (EDTA) containing vacutainers were used to collect approximately 3 ml of blood samples from each study participants. Genomic DNA isolation was performed utilizing QIAmp DNA blood mini kit (Catalog no. 51104, Qiagen, Valencia, CA, United States) as per the manufacturer’s instructions. Spectrophotometric quantitation and purity of the extracted DNA was done on NanoDrop 8000 (Thermo Scientific, Waltham, MA, United States).

### SNP Selection and Genotyping

A total of four single nucleotide polymorphism in NOTCH1, NOTCH2, NOTCH3 and NOTCH4 receptor genes were selected from previous literature [18–24]. TaqMan allelic discrimination assays were used to genotype the SNPs based on Livak’s method [25]. Briefly, for each sample, 20 ng of purified genomic DNA was mixed with 5.0 µl of 2× TaqMan genotyping Master Mix (Catalog no. 4371355, Applied Biosystems, Foster City, CA, United States) and 0.25 µl of 40× TaqMan SNP genotyping assay (Catalog no. 4351379, Assay ID: C____189,059_10; C__31617470_30; C___7494157_10; C__27523194_10, Thermo Fisher Scientific, United States) containing the primers and probe in a total volume of 10 µL performed in Fast Optical 96-Well Reaction Plate (Catalog no. 4346906, Applied Biosystems, Foster City, CA, United States). The genotypes were determined by endpoint reading on QuantStudio 7 Flex Real Time PCR system (Applied Biosystems, Foster City, CA, United States). The instrument was programmed as follows: pre-read at 60°C for 30 s, polymerase activation at 95°C for 10 min, 40 cycles of denaturation at 95°C for 15 s and annealing/extension at 60°C for 1 min followed by post-read at 60°C for 30 s. TaqMan Genotyper Software version 1.4 was used to automatically analyze the data and make the genotype calls.

### Statistical Analysis

Frequencies for the three genotypes and alleles for each SNP were computed and tests for deviation from Hardy-Weinberg equilibrium and tests for association were performed using publicly available web-based tool at https://ihg.helmholtz-muenchen.de/cgi-bin/hw/hwa1.pl. Genetic association of each SNP with breast and colorectal cancer were determined by case-control comparisons using the chi-square test and odds ratios (OR), and 95% CI. A *p*-value of ≤ 0.05 was considered as significant. Additionally, since we analyzed 4 SNPs in total, Bonferroni’s correction for multiple comparison was applied with an *α* = 0.0125 considered as significant.

## Results

We examined the genotypes of four germline SNPs residing in NOTCH1 - rs3124591, NOTCH2 - rs11249433, NOTCH3 - rs1043994, and NOTCH4 - rs3830041 to determine their association with breast and colorectal cancer risk in Saudi Arabian patients. The rs3124591 is a 3 Prime UTR variant in NOTCH1, rs11249433 resides in a linkage disequilibrium block neighboring NOTCH2, rs1043994 is a synonymous variant coding for alanine at protein position 202 of NOTCH3 and rs3830041 is an intronic variant in NOTCH4 receptor.

### Association of Genetic Variants in NOTCH Receptors With Breast and Colorectal Cancers

#### Breast Cancer

The distribution of genotypes for all the four SNPs in control group as well as NOTCH3 rs1043994 and NOTCH4 rs3830041 in breast cancers followed Hardy-Weinberg equilibrium while SNPs rs3124591 in NOTCH1 (*p* = 0.046275) and NOTCH2 related rs11249433 (*p* = 0.000095) in breast cancers deviated from Hardy-Weinberg equilibrium (Table 1). Clinicopathological and demographic data along with genotypes of the examined SNPs for each breast cancer cases and controls are presented in Supplementary Table S1.

**TABLE 1:** Test for deviation from Hardy-Weinberg equilibrium.

SNP ID	Genotype	Controls *n* (frequency)	HWE *p*-value	Cancer *n* (frequency)	HWE *p*-value
**Breast cancer**					
rs3124591	CC	52 (0.74)	0.217048	142 (0.75)	**0.046275**
	CT	18 (0.26)		48 (0.25)	
	TT	00 (0.00)		00 (0.00)	
rs11249433	GG	19 (0.14)	0.730739	14 (0.08)	**0.000095**
	GA	65 (0.49)		112 (0.60)	
	AA	49 (0.37)		59 (0.32)	
rs1043994	GG	64 (0.5)	0.053619	103 (0.566)	0.507910
	GA	59 (0.46)		70 (0.385)	
	AA	05 (0.04)		09 (0.049)	
rs3830041	CC	57 (0.42)	0.958591	91 (0.49)	0.445045
	CT	61 (0.46)		75 (0.40)	
	TT	16 (0.12)		20 (0.11)	
**Colorectal cancer**					
rs3124591	CC	82 (0.80)	0.249306	74 (0.77)	0.204808
	CT	21 (0.20)		22 (0.23)	
	TT	00 (00)		00 (00)	
rs11249433	GG	13 (0.09)	0.179768	16 (0.11)	0.250366
	GA	70 (0.50)		72 (0.51)	
	AA	56 (0.40)		53 (0.38)	
rs1043994	GG	84 (0.60)	0.117103	76 (0.57)	**0.019035**
	GA	52 (0.37)		56 (0.42)	
	AA	03 (0.02)		2 (0.01)	
rs3830041	CC	69 (0.50)	0.284159	75 (0.53)	0.095852
	CT	54 (0.39)		50 (0.35)	
	TT	16 (0.12)		16 (0.11)	

HWE, Hardy-Weinberg equilibrium; *p* ≤ 0.05 was considered significant and are depicted in bold and deviated from HWE.

The distributions of genotype and allele frequencies of the examined SNPs are shown in Table 2. In the overall analysis, only rs11249433 SNP that is in linkage disequilibrium with NOTCH2 exhibited statistically significant association with breast cancer susceptibility. In breast cancer cases the GA genotype was detected at a significantly higher proportion of 60% compared to 49% in the control group. Women that harbor heterozygous GA genotype for this SNP were at 2.3-fold higher risk of developing carcinoma of the breast compared to those having GG genotype (OR = 2.338, χ^2^ = 5.04, *p* = 0.02478). The other three SNPs, rs3124591 (NOTCH1), rs1043994 (NOTCH3), and rs3830041 (NOTCH4) were not significantly associated with the predisposition of breast carcinoma in the overall analysis (Table 2).

**TABLE 2:** NOTCH receptors SNPs genotype and allele frequencies in breast cancer cases and control population.

SNP ID	Genotype	Controls *n* (frequency)	Breast cancer *n* (frequency	OR (95%CI)	χ^2^ -value	*p*-value*
rs3124591, NOTCH1	CC	52 (0.74)	142 (0.75)	Ref		
	CT	18 (0.26)	48 (0.25)	0.977 (0.521–1.830)	0.01	0.94090
	TT	00 (0.00)	00 (0.00)	0.368 (0.007–18.806)	na	1.00000
	Allele					
	C	122 (0.87)	332 (0.87)	Ref		
	T	18 (0.13)	48 (0.13)	0.980 (0.549–1.750)	0.00	0.94536
rs11249433, NOTCH2	GG	19 (0.14)	14 (0.08)	Ref		
	GA	65 (0.49)	112 (0.60)	2.338 (1.099–4.975)	5.04	**0.02478**
	AA	49 (0.37)	59 (0.32)	1.634 (0.744–3.591)	1.51	0.21943
	Allele					
	G	103 (0.39)	140 (0.38)	Ref		
	A	163 (0.61)	230 (0.62)	1.038 (0.751–1.435)	0.05	0.82096
rs1043994, NOTCH3	GG	64 (0.5)	103 (0.566)	Ref		
	GA	59 (0.46)	70 (0.385)	0.737 (0.463–1.175)	1.65	0.19939
	AA	05 (0.04)	09 (0.049)	1.118 (0.359–3.486)	0.04	0.84690
	Allele					
	G	187 (0.73)	276 (0.76)	Ref		
	A	69 (0.27)	88 (0.24)	0.864 (0.599–1.246)	0.61	0.43364
rs3830041, NOTCH4	CC	57 (0.42)	91 (0.49)	Ref		
	CT	61 (0.46)	75 (0.40)	0.770 (0.480–1.236)	1.17	0.27882
	TT	16 (0.12)	20 (0.11)	0.783 (0.375–1.635)	0.43	0.51417
	Allele					
	C	175 (0.65)	257 (0.69)	Ref		
	T	93 (0.35)	115 (0.31)	0.842 (0.603–1.176)	1.02	0.31284

OR 95% CI, Odds Ratio and 95% Confidence Interval; na, not analyzable.

* *p* ≤ 0.05 was considered significant and are depicted in bold.

#### Colorectal Cancer

Pathologically confirmed colorectal cancer cases and age as well as gender-matched controls from Saudi Arabian population without prior history of cancer were examined to find an association of genetic variants in NOTCH receptors and susceptibility of CRC. The genotypes distributions of all the SNPs were in accordance with Hardy-Weinberg equilibrium in control population. In CRC patients, genotypes of all SNPs except NOTCH3 rs1043994 followed Hardy-Weinberg equilibrium (Table 1). The clinicopathological and demographic data for each colorectal cancers and controls along with genotypes of analyzed SNPs are presented in Supplementary Table S2.

The genotypes and allele frequencies in CRCs and controls are shown in Table 3. The genotypic and allelic frequencies in CRC patients and control group were not significantly different and hence these SNPs in the NOTCH receptors were not associated with the susceptibility of colorectal cancers in our cohort in the overall analysis (Table 3).

**TABLE 3:** NOTCH receptors SNPs genotype and allele frequencies in colorectal cancer cases and control population.

SNP ID	Genotype	Controls *n* (frequency)	CRC *n* (frequency)	OR (95%CI)	χ^2^ -value	*p*-value*
rs3124591, NOTCH1	CC	82 (0.80)	74 (0.77)	Ref		
	CT	21 (0.20)	22 (0.23)	1.161 (0.591–2.281)	0.19	0.66499
	TT	00 (00)	00 (00)	1.107 (0.022–56.510)	na	1.00000
	Allele					
	C	185 (0.90)	170 (0.89)	Ref		
	T	21 (0.10)	22 (0.11)	1.140 (0.605–2.147)	0.16	0.68477
rs11249433, NOTCH2	GG	13 (0.09)	16 (0.11)	Ref		
	GA	70 (0.50)	72 (0.51)	0.836 (0.375–1.864)	0.19	0.66086
	AA	56 (0.40)	53 (0.38)	0.769 (0.338–1.750)	0.39	0.53077
	Allele					
	G	96 (0.35)	104 (0.37)	Ref		
	A	182 (0.65)	178 (0.63)	0.903 (0.639–1.276)	0.34	0.56221
rs1043994, NOTCH3	GG	84 (0.60)	76 (0.57)	Ref		
	GA	52 (0.37)	56 (0.42)	1.190 (0.730–1.940)	0.49	0.48457
	AA	03 (0.02)	2 (0.01)	0.737 (0.120–4.529)	0.11	0.74081
	Allele					
	G	220 (0.79)	208 (0.78)	Ref		
	A	58 (0.21)	60 (0.22)	1.094 (0.728–1.645)	0.19	0.66521
rs3830041, NOTCH4	CC	69 (0.50)	75 (0.53)	Ref		
	CT	54 (0.39)	50 (0.35)	0.852 (0.514–1.411)	0.39	0.53349
	TT	16 (0.12)	16 (0.11)	0.920 (0.428–1.979)	0.05	0.83107
	Allele					
	C	192 (0.69)	200 (0.71)	Ref		
	T	86 (0.31)	82 (0.29)	0.915 (0.638–1.314)	0.23	0.63157

CRC, colorectal cancer; OR 95% CI, odds ratio and 95% Confidence Interval; na, not analyzable.

* *p* ≤ 0.05 was considered significant and are depicted in bold.

### Genetic Variants in NOTCH Receptors and Age of Onset of Breast and Colorectal Cancers

#### Breast Cancer

To determine the association between genetic variants in NOTCH receptors and age of onset of breast cancer, we segregated the cases and control groups based on the median age of breast cancer diagnosis as ≤53 years and >53 years. As observed for the overall analysis, the rs11249433 (NOTCH2) SNP was found to be significantly associated with early onset (≤53 years of age at diagnosis) of breast cancer. The GA genotype of NOTCH2 related rs11249433 SNP conferred about 4.7 fold higher risk of developing carcinoma of the breast before or till the age of 53 years relative to those having GG genotype (OR = 4.692, χ^2^ = 7.26, *p* = 0.00707) (Table 4). This association was maintained even after Bonferroni’s correction for multiple comparisons. The other variants, rs3124591 (NOTCH1), rs1043994 (NOTCH3), and rs3830041 (NOTCH4) did not influence the early onset of breast malignancies in our patients. Similarly, the distribution of genotype and allele frequencies of all the four SNPs were comparable in controls and breast cancer patients whose age at the time of diagnosis was >53 years and hence were not associated with the late onset of the disease (Table 4).

**TABLE 4:** NOTCH receptors SNPs genotype and allele frequencies in breast cancer cases and control population based on age.

SNP ID	Genotype	Controls *n* (frequency)	Breast cancer *n* (frequency)	OR (95%CI)	χ^2^ -value	*p*-value*
**≤53 years**						
rs3124591, NOTCH1	CC	39 (0.74)	72 (0.735)	Ref		
	CT	14 (0.26)	26 (0.265)	1.006 (0.472–2.146)	0.00	0.98775
	TT	00 (0.00)	00 (0.00)	0.545 (0.011–27.988)	na	1.00000
	Allele					
	C	92 (0.87)	170 (0.87)	Ref		
	T	14 (0.13)	26 (0.13)	1.005 (0.500–2.019)	0.00	0.98872
rs11249433, NOTCH2	GG	12 (0.15)	04 (0.04)	Ref		
	GA	39 (0.49)	61 (0.64)	4.692 (1.412–15.592)	7.26	**0.00707**
	AA	29 (0.36)	31 (0.32)	3.207 (0.928–11.079)	3.62	0.05724
	Allele					
	G	63 (0.39)	69 (0.36)	Ref		
	A	97 (0.61)	123 (0.64)	1.158 (0.751–1.785)	0.44	0.50712
rs1043994, NOTCH3	GG	38 (0.47)	58 (0.604)	Ref		
	GA	39 (0.49)	34 (0.354)	0.571 (0.309–1.057)	3.20	0.07350
	AA	3 (0.04)	4 (0.042)	0.874 (0.185–4.123)	0.03	0.86436
	Allele					
	G	115 (0.72)	150 (0.78)	Ref		
	A	45 (0.28)	42 (0.22)	0.716 (0.440–1.163)	1.83	0.17587
rs3830041, NOTCH4	CC	30 (0.37)	48 (0.495)	Ref		
	CT	40 (0.49)	41 (0.423)	0.641 (0.341–1.204)	1.92	0.16550
	TT	11 (0.14)	8 (0.082)	0.455 (0.164–1.259)	2.36	0.12412
	Allele					
	C	100 (0.62)	137 (0.71)	Ref		
	T	62 (0.38)	57 (0.29)	0.671 (0.431–1.045)	3.14	0.07661
**>53 years**						
rs3124591, NOTCH1	CC	13 (0.76)	70 (0.76)	Ref		
	CT	04 (0.24)	22 (0.24)	1.021 (0.302–3.455)	0.00	0.97280
	TT	00 (0.00)	00 (0.00)	0.191 (0.004–10.075)	na	1.00000
	Allele					
	C	30 (0.88)	162 (0.88)	Ref		
	T	04 (0.12)	22 (0.12)	1.019 (0.328–3.167)	0.00	1.00000
rs11249433, NOTCH2	GG	07 (0.13)	10 (0.112)	Ref		
	GA	26 (0.49)	51 (0.573)	1.373 (0.469–4.024)	0.34	0.56235
	AA	20 (0.38)	28 (0.315)	0.980 (0.319–3.014)	0.00	0.97188
	Allele					
	G	40 (0.38)	71 (0.40)	Ref		
	A	66 (0.62)	107 (0.60)	0.913 (0.557–1.497)	0.13	0.71926
rs1043994, NOTCH3	GG	26 (0.54)	45 (0.52)	Ref		
	GA	20 (0.42)	36 (0.42)	1.040 (0.502–2.157)	0.01	0.91605
	AA	02 (0.04)	05 (0.06)	1.444 (0.261–7.982)	0.18	0.67192
	Allele					
	G	72 (0.75)	126 (0.73)	Ref		
	A	24 (0.25)	46 (0.27)	1.095 (0.618–1.941)	0.10	0.75530
rs3830041, NOTCH4	CC	27 (0.51)	43 (0.483)	Ref		
	CT	21 (0.40)	34 (0.382)	1.017 (0.492–2.102)	0.00	0.96454
	TT	05 (0.09)	12 (0.135)	1.507 (0.478–4.754)	0.49	0.48236
	Allele					
	C	75 (0.71)	120 (0.674)	Ref		
	T	31 (0.29)	58 (0.326)	1.169 (0.693–1.973)	0.34	0.55740

OR 95% CI, odds ratio and 95% Confidence Interval; na, not analyzable.

* *p* ≤ 0.05 was considered significant and are depicted in bold.

#### Colorectal Cancer

Colorectal cancer cases and controls were segregated according to the median age at the time of disease diagnosis as ≤58 years and >58 years. None of the four SNPs examined in the NOTCH receptors were found to be significantly associated with the age of onset of colorectal cancers (Table 5).

**TABLE 5:** NOTCH receptors SNPs genotype and allele frequencies in colorectal cancer cases and control population based on age.

SNP ID	Genotype	Controls *n* (frequency)	CRC *n* (frequency)	OR (95%CI)	χ^2^ -value	*p*-value*
**≤58 years**						
rs3124591, NOTCH1	CC	55 (0.76)	47 (0.77)	Ref		
	CT	17 (0.24)	14 (0.23)	0.964 (0.430–2.161)	0.01	0.92849
	TT	00 (0.00)	00 (0.00)	1.168 (0.023–60.020)	na	1.00000
	Allele					
	C	127 (0.88)	108 (0.89)	Ref		
	T	17 (0.12)	14 (0.11)	0.968 (0.456–2.055)	0.01	0.93336
rs11249433, NOTCH2	GG	07 (0.09)	08 (0.11)	Ref		
	GA	36 (0.49)	36 (0.50)	0.875 (0.287–2.667)	0.06	0.81428
	AA	31 (0.42)	28 (0.39)	0.790 (0.254–2.461)	0.17	0.68435
	Allele					
	G	50 (0.34)	52 (0.36)	Ref		
	A	98 (0.66)	92 (0.64)	0.903 (0.558–1.461)	0.17	0.67664
rs1043994, NOTCH3	GG	42 (0.57)	37 (0.54)	Ref		
	GA	29 (0.39)	32 (0.46)	1.253 (0.641–2.446)	0.44	0.50930
	AA	03 (0.04)	00 (0.00)	0.162 (0.008–3.237)	2.56	0.10958
	Allele					
	G	113 (0.76)	106 (0.77)	Ref		
	A	35 (0.24)	32 (0.23)	0.975 (0.564–1.686)	0.01	0.92683
rs3830041, NOTCH4	CC	36 (0.49)	34 (0.47)	Ref		
	CT	31 (0.42)	27 (0.38)	0.922 (0.459–1.852)	0.05	0.81984
	TT	07 (0.09)	11 (0.15)	1.664 (0.578–4.789)	0.90	0.34250
	Allele					
	C	103 (0.70)	95 (0.66)	Ref		
	T	45 (0.30)	49 (0.34)	1.181 (0.722–1.930)	0.44	0.50773
**>58 years**						
rs3124591, NOTCH1	CC	27 (0.87)	27 (0.77)	Ref		
	CT	04 (0.13)	08 (0.23)	2.000 (0.538–7.438)	1.09	0.29538
	TT	00 (00)	00 (0.00)	1.000 (0.019–52.217)	na	1.00000
	Allele					
	C	58 (0.94)	62 (0.89)	Ref		
	T	04 (0.06)	08 (0.11)	1.871 (0.535–6.547)	0.99	0.32086
rs11249433, NOTCH2	GG	06 (0.09)	08 (0.12)	Ref		
	GA	34 (0.52)	36 (0.52)	0.794 (0.250–2.527)	0.15	0.69594
	AA	25 (0.38)	25 (0.36)	0.750 (0.227–2.477)	0.22	0.63644
	Allele					
	G	46 (0.35)	52 (0.38)	Ref		
	A	84 (0.65)	86 (0.62)	0.906 (0.551–1.490)	0.15	0.69644
rs1043994, NOTCH3	GG	42 (0.65)	39 (0.60)	Ref		
	GA	23 (0.35)	24 (0.37)	1.124 (0.548–2.306)	0.10	0.75044
	AA	00 (00)	02 (0.03)	5.380 (0.250–115.55)	2.10	0.14736
	Allele					
	G	107 (0.82)	102 (0.78)	Ref		
	A	23 (0.18)	28 (0.22)	1.277 (0.691–2.361)	0.61	0.43486
rs3830041, NOTCH4	CC	33 (0.51)	41 (0.59)	Ref		
	CT	23 (0.35)	23 (0.33)	0.805 (0.385–1.683)	0.33	0.56389
	TT	09 (0.14)	05 (0.07)	0.447 (0.137–1.463)	1.83	0.17616
	Allele					
	C	89 (0.68)	105 (0.76)	Ref		
	T	41 (0.32)	33 (0.24)	0.682 (0.398–1.169)	1.95	0.16287

CRC, colorectal cancer; OR 95% CI. odds ratio and 95% Confidence Interval; na, not analyzable.

* *p* ≤ 0.05 was considered significant and are depicted in bold.

### Association of SNPs in NOTCH Receptors With Colorectal Cancer Based on Gender

In order to evaluate whether gender played any role in the association of NOTCH1-rs3124591, NOTCH2-rs11249433, NOTCH3-rs1043994, and NOTCH4-rs3830041 with colorectal cancers, the study subjects were grouped as males and females for counting the genotype and allele frequencies. The distributions of these frequencies are depicted in Table 6. The rs1043994 SNP in the NOTCH3 receptor showed statistically significant association with colorectal cancers in males. The GA heterozygous males were about 2-fold higher risk of developing CRC relative to those with homozygous GG genotype (OR = 1.971, χ^2^ = 4.01, *p* = 0.04514) (Table 6). The other three SNPs were not associated with CRCs in males. Moreover, we did not observe any of the four SNPs to be associated with CRCs in females in our population.

**TABLE 6:** NOTCH receptors SNPs genotype and allele frequencies in colorectal cancer cases and control population based on gender.

SNP ID	Genotype	Controls *n* (frequency)	CRC *n* (frequency)	Or (95%CI)	χ^2^ -value	*p*-value*
**Male**						
rs3124591, NOTCH1	CC	45 (0.9)	39 (0.76)	Ref		
	CT	05 (0.1)	12 (0.24)	2.769 (0.896–8.555)	3.30	0.06922
	TT	00 (00)	0 (0)	1.152 (0.022–59.409)	na	1.00000
	Allele					
	C	95 (0.95)	90 (0.88)	Ref		
	T	05 (0.05)	12 (0.12)	2.533 (0.858–7.478)	3.00	0.08337
rs11249433, NOTCH2	GG	08 (0.11)	11 (0.13)	Ref		
	GA	35 (0.48)	41 (0.49)	0.852 (0.308–2.354)	0.10	0.75718
	AA	30 (0.41)	31 (0.37)	0.752 (0.266–2.126)	0.29	0.58971
	Allele					
	G	51 (0.35)	63 (0.38)	Ref		
	A	95 (0.65)	103 (0.62)	0.878 (0.553–1.394)	0.31	0.58040
rs1043994, NOTCH3	GG	49 (0.67)	40 (0.51)	Ref		
	GA	23 (0.32)	37 (0.47)	1.971 (1.011–3.841)	4.01	**0.04514**
	AA	01 (0.01)	1 (0.01)	1.225 (0.074–20.207)	0.02	0.88698
	Allele					
	G	121 (0.83)	117 (0.75)	Ref		
	A	25 (0.17)	39 (0.25)	1.613 (0.919–2.832)	2.80	0.09417
rs3830041, NOTCH4	CC	39 (0.53)	45 (0.54)	Ref		
	CT	28 (0.38)	34 (0.41)	1.052 (0.545–2.034)	0.02	0.87927
	TT	6 (0.08)	4 (0.05)	0.578 (0.152–2.197)	0.66	0.41672
	Allele					
	C	106 (0.73)	124 (0.75)	Ref		
	T	40 (0.27)	42 (0.25)	0.898 (0.542–1.487)	0.18	0.67470
**Female**						
rs3124591, NOTCH1	CC	37 (0.7)	35 (0.78)	Ref		
	CT	16 (0.3)	10 (0.22)	0.661 (0.265–1.650)	0.79	0.37338
	TT	0 (0)	0 (0)	1.056 (0.020–54.681)	na	1.00000
	Allele					
	C	90 (0.85)	80 (0.89)	Ref		
	T	16 (0.15)	10 (0.11)	0.703 (0.302–1.638)	0.67	0.41263
rs11249433, NOTCH2	GG	5 (0.08)	5 (0.09)	Ref		
	GA	35 (0.53)	31 (0.53)	0.886 (0.234–3.351)	0.03	0.85806
	AA	26 (0.39)	22 (0.38)	0.846 (0.216–3.308)	0.06	0.81009
	Allele					
	G	45 (0.34)	41 (0.35)	Ref		
	A	87 (0.66)	75 (0.65)	0.946 (0.560–1.598)	0.04	0.83600
rs1043994, NOTCH3	GG	35 (0.53)	36 (0.64)	Ref		
	GA	29 (0.44)	19 (0.34)	0.637 (0.303–1.338)	1.42	0.23260
	AA	2 (0.03)	1 (0.02)	0.486 (0.042–5.606)	0.35	0.55558
	Allele					
	G	99 (0.75)	91 (0.81)	Ref		
	A	33 (0.25)	21 (0.19)	0.692 (0.374–1.283)	1.37	0.24123
rs3830041, NOTCH4	CC	30 (0.45)	30 (0.52)	Ref		
	CT	26 (0.39)	16 (0.28)	0.615 (0.276–1.373)	1.41	0.23437
	TT	10 (0.15)	12 (0.21)	1.200 (0.450–3.197)	0.13	0.71522
	Allele					
	C	86 (0.65)	76 (0.66)	Ref		
	T	46 (0.35)	40 (0.34)	0.984 (0.583–1.662)	0.00	0.95185

CRC, colorectal cancer; OR 95% CI, odds ratio and 95% Confidence Interval; na, not analyzable.

* *p* ≤ 0.05 was considered significant and are depicted in bold.

## Discussion

Notch signaling pathway is highly conserved molecular cell signaling pathway which plays important roles in proliferation, differentiation, cell fate specification, homeostasis and angiogenesis. Additionally, notch signaling is considered as one of the most common pathway implicated in cancer metastasis [26]. Several investigations have led to the conclusion that alterations in notch signaling pathway are associated with the development of various cancers including colon [15] and breast [27]. Most of the diseases including cancer are result of the interaction between genetic and environmental factors. A number of studies have indicated that genetic variation contributes in part toward the susceptibility of common diseases such as diabetes and cancer [28–30]. The identification of genetic variation associated with cancer may assist in revealing the underlying pathophysiological processes in the initiation and progression of the disease. There has been an increased interest in the most common functional germline polymorphisms on clinical outcomes for patients with cancer. The presence of genetic variation in the human genome can be found in different forms and frequencies throughout the genome. Among these forms are single nucleotide polymorphisms which are considered as the main source of genetic variation in human genome and account for about 90 percent of all human genetic variations. They occur roughly every 100–300 bases [31].

Several studies have shown that SNPs in NOTCH receptors are linked to risk and prognosis of a number of diseases. For examples, SNPs in NOTCH1 and NOTCH2 are associated with risk of breast carcinoma [19, 22]. Genetic variants in NOTCH3 gene have been shown to be associated with cerebral small vessel disease [32], while NOTCH4 variants linked to Alzheimer's disease [33]. In the present study, we evaluated for the first time the association of NOTCH1, rs3124591; NOTCH2, rs11249433; NOTCH3, rs1043994, and NOTCH4, rs3830041 SNPs with the susceptibility of breast and colorectal cancers in Saudi population.

In the overall analysis, except for rs11249433 which is in close proximity to NOTCH2 gene that showed significant association with breast cancer, none of the other SNPs were found to confer increased risk either in breast or colorectal cancers in our population. Investigations on the association of NOTCH1 rs3124591, NOTCH3 rs1043994, and NOTCH4 rs3830041 with the risk of human cancers are rare, however, several studies have shown a link between NOTCH2 related rs11249433 and increased risk of breast cancer especially in women of European ancestry [18–21]. The rs11249433 variant is located in the pericentromeric region at 1p11.2 and NOTCH2, a transmembrane coding gene and FCGR1B (low-affinity Fc gamma receptor family) are the nearest genes to this SNP. Hunter’s group conducted a large genome-wide scan plus two stages of follow-up in 10,263 controls and 9,335 cases and found conclusive statistically significant association of NOTCH2 related rs11249433 with breast cancer [18]. They further investigated 6,386 cases for which estrogen receptor (ER) status was available to reveal that this association was more apparent for ER+ relative to ER-breast tumor. Similar association of NOTCH2-rs11249433 with ER status was not found in our breast cancer cohort. Since cancer-associated SNPs have been shown to be linked to alterations in gene expression, Prokunina-Olsson and colleagues examined and reported that the risk genotypes of rs11249433 have a positive association with NOTCH2 mRNA expression in TP53 wild-type/ER+ breast cancers [19]. Campa et al confirmed the association of NOTCH2 rs11249433 with breast cancer risk but did not find statistically significant interaction with nine established risk factors such as age at menarche, parity, age at menopause, use of hormone replacement therapy, family history, height, body mass index, smoking status, and alcohol consumption [20]. Furthermore, a comprehensive meta-analysis comprising 90,154 cases and 137,238 controls was conducted by Wu et al to assess the relationship between the NOTCH2 rs11249433 polymorphism and breast cancer susceptibility. Their analysis showed that rs11249433 polymorphism poses significant risk in Caucasians but not in Africans and East Asians [21]. The lack of significant association between NOTCH2 rs11249433 and breast cancer risk in Chinese population as well shown by Jiang et al suggest ethnic specificity for this locus in conferring disease susceptibility [34]. Our finding of a significant association of NOTCH2 rs11249433 with the risk of breast cancer suggests that Saudi population may be closer to the Europeans than to Africans or Asians in terms of genetic susceptibility to breast cancer. Moreover, it has been demonstrated that the NOTCH2 rs11249433 exhibited a stronger association with the development of breast cancer especially with ER-positive tumors compared to ER-negative tumors [18, 35]. However, Campa et al did not observe similar association of this SNP with risk of breast cancer by ER status [20] Another study indicated that the NOTCH2 rs11249433 was associated with the risk of breast cancer for patients who are BRCA2 mutation carrier, but was not associated with the risk of breast cancer for BRCA1 mutation carriers [36]. In our comparison by age at diagnosis, we observed strong association of NOTCH2 rs11249433 with increased risk of early onset of breast cancer. The GA heterozygotes were at about 5-fold increased risk of developing breast cancer at younger age compared to those harboring GG genotype. Similar association of this SNP with risk of breast cancer pertaining the age at diagnosis was not observed in women of European ancestry [18, 20]. This discrepancy could be due to other environmental as well as genetic factors and need further investigations.

Our data showed that rs1043994 in NOTCH3 although not significantly associated with breast or colorectal cancer in the overall analysis was having a statistically significant association with colorectal cancers in males. The GA heterozygote males of this SNP were at approximately 2-fold higher risk of developing colorectal cancers compared to GG homozygotes. Colorectal cancer is the predominant cancer in Saudi Arabian males. As cancer risk can be influenced by differential gene expression pattern between men and women as a result of differences in their hormonal and genetic factors, the association of colorectal cancer in men could be attributed to NOTCH3 - rs1043994 variants. Gender related differences in the prognosis of several cancers including colorectal cancer have been reported [37–41]. A link between genetic polymorphism and overall survival in colorectal cancer patients based on gender has been demonstrated in earlier studies [42, 43]. Yagci et al indicated that NOTCH3 rs1043994 is associated with the risk of developing lung cancer in patients of Turkish origin [24]. Associations of NOTCH3-rs1043994 synonymous variant with lacunar infarction and migraine have also been reported in Chinese and German patients, respectively [44, 45].

In Chinese population, while the association of NOTCH2-rs11249433 and NOTCH3-rs1043994 was lacking with breast cancer risk, NOTCH1-rs3124591 was significantly associated with invasive ductal carcinoma and ductal carcinoma *in situ* [22]. Furthermore, a positive correlation between TC genotype of NOTCH1-rs3124591 and high notch1 protein expression in ductal carcinoma *in situ* but not in invasive ductal carcinoma was observed [22]. Our data did not show any significant association of NOTCH1-rs3124591 with either CRC or breast cancer. Besides, NOTCH1-rs3124591 is also significantly correlated with nephrotic syndrome risk and alteration in its sensitivity to hormone in Chinese population [46]. A recent study by Yu et al demonstrated that Chinese patients carrying the TT genotype of NOTCH4-rs3830041 had poorer overall survival in contrast to those carrying TC/CC genotype and concluded that rs3830041 variant is an independent predictive marker for prognosis in hepatitis B virus-related hepatocellular carcinoma patients [23]. However, we did not find significant association of NOTCH4-rs3830041 with risk of breast or colorectal cancer in our population.

While there are few reports in the literature on the correlation of the four NOTCH receptor SNPs that we examined on breast cancer, this is the first study to screen these SNPs in colorectal cancers. Screening of larger population of different ethnicity validating our findings on the association of NOTCH2-rs11249433 with breast cancer particularly in younger women and NOTCH3-rs1043994 with colorectal cancer in men would prove beneficial in utilizing these variants as genetic markers for early diagnosis and management of these malignancies.

## Data Availability

The original contributions presented in the study are included in the article/Supplementary Material, further inquiries can be directed to the corresponding author.
